# Computer-aided diagnosis for (^123^I)FP-CIT imaging: impact on clinical reporting

**DOI:** 10.1186/s13550-018-0393-5

**Published:** 2018-05-08

**Authors:** Jonathan Christopher Taylor, Charles Romanowski, Eleanor Lorenz, Christine Lo, Oliver Bandmann, John Fenner

**Affiliations:** 1 Sheffield Teaching Hospitals NHS Foundation Trust, Nuclear Medicine, I-floor, Royal Hallamshire Hospital, Glossop road, Sheffield, S10 2JF UK; 2 Sheffield Teaching Hospitals NHS Foundation Trust, Radiology, Royal Hallamshire Hospital, Glossop road, Sheffield, S10 2JF UK; 3 Oxford Parkinson’s Disease Centre, University of Oxford, Le Gros Clark Building, South Parks road, Oxford, OX1 3QX UK; 4 Department of Neuroscience, Sheffield Institute for Translational Neuroscience, University of Sheffield, 385a Glossop road, Sheffield, S10 2HQ UK; 5 Insigneo, Infection Immunity and Cardiovascular Disease, University of Sheffield, Royal Hallamshire Hospital, Glossop road, Sheffield, S10 2JF UK

**Keywords:** (^123^I)FP-CIT, Machine learning, Support vector machine, Computer-aided diagnosis

## Abstract

**Background:**

For (^123^I)FP-CIT imaging, a number of algorithms have shown high performance in distinguishing normal patient images from those with disease, but none have yet been tested as part of reporting workflows. This study aims to evaluate the impact on reporters’ performance of a computer-aided diagnosis (CADx) tool developed from established machine learning technology.

Three experienced (^123^I)FP-CIT reporters (two radiologists and one clinical scientist) were asked to visually score 155 reconstructed clinical and research images on a 5-point diagnostic confidence scale (read 1). Once completed, the process was then repeated (read 2). Immediately after submitting each image score for a second time, the CADx system output was displayed to reporters alongside the image data. With this information available, the reporters submitted a score for the third time (read 3). Comparisons between reads 1 and 2 provided evidence of intra-operator reliability, and differences between reads 2 and 3 showed the impact of the CADx.

**Results:**

The performance of all reporters demonstrated a degree of variability when analysing images through visual analysis alone. However, inclusion of CADx improved consistency between reporters, for both clinical and research data. The introduction of CADx increased the accuracy of the radiologists when reporting (unfamiliar) research images but had less impact on the clinical scientist and caused no significant change in accuracy for the clinical data.

**Conclusions:**

The outcomes for this study indicate the value of CADx as a diagnostic aid in the clinic and encourage future development for more refined incorporation into clinical practice.

## Background

(^123^I)FP-CIT (ioflupane) single-photon emission computed tomography (SPECT) is routinely used for assessment and differential diagnosis of patients with Parkinsonian syndromes. (^123^I)FP-CIT SPECT is pathological in patients with any neurodegenerative form of Parkinsonism, including not only classical Parkinson’s disease (PD), but also atypical Parkinsonian disorders such as multiple system atrophy (MSA) or progressive supranuclear palsy (PSP). It is normal in patients with non-neurodegenerative movement disorders such as drug-induced Parkinsonism or essential tremor. In recent years, different automated classification algorithms have been developed which aim to accurately separate these images into binary diagnostic groups: either with disease or without disease. Many such classifiers are based on machine learning approaches. For instance, Palumbo et al. created classifiers based on support vector machines (SVMs) and neural networks to separate patients with PD from those without [1]. Huertas-Fernández et al. developed and evaluated models based on logistic regression, linear discriminant analysis and SVMs to differentiate between patients with PD and vascular Parkinsonism [2]. A summary of recently published machine learning algorithms for (^123^I)FP-CIT classification is presented in recent work by Taylor [3]. Performance figures from many of these classification tools appear to be impressive, with accuracies in excess of 95% commonly reported [3]. However, it is not yet clear whether such algorithms provide benefits in the clinic in terms of increased diagnostic accuracy or consistency as compared to standard reporting procedures.

The likely use for automated classifiers in (^123^I)FP-CIT imaging, and for other areas of nuclear medicine in the near term, is either as an independent assistant to the radiologist or as a training/audit tool, whereby reporter performance is compared to an independent assessment. In this study, the first scenario is considered, where the classifier performs the role of a second reporter, giving a second opinion on image appearances, which may influence the reporter’s final diagnostic decision. Using classification algorithms in this way is often referred to as computer-aided diagnosis (CADx).

In (^123^I)FP-CIT, assistive reporting software, in the form of semi-quantification, is already established. Here, relative uptake in striatal regions of interest is compared to an area of non-specific uptake and displayed alongside reference values. This provides radiologists with a parameter that can be related to the likelihood of disease being present. Use of such tools has been shown to increase consistency between reporters and improve confidence [4–9]. However, semi-quantification is a limited diagnostic tool. Firstly, standalone performance has been shown to be inferior to that of even relatively simple machine learning algorithms [3]. Furthermore, semi-quantification software may provide large numbers of uptake ratios, each with their own associated normal range. It can be challenging to interpret each of these sets of figures to give an overall opinion on image appearances. Machine learning tools, on the other hand, can be tuned to provide just a single output related to the probability of disease being present. Therefore, there is potential for CADx systems based on machine learning algorithms to provide more effective assistance to reporters, to give improved reporting performance. To date, there has been no exploration of the potential for automated classifiers in clinical, computer-aided (^123^I)FP-CIT reporting. This limits the usefulness of this approach.

The following study aims to address this issue by examining the performance of experienced reporters, with and without assistance from an automated classifier. Although the automated classifier is based on a particular machine learning methodology, results are likely to be reflective of the potential benefits of any highly performing binary classification tool and therefore provide insights into the general impact of CADx on (^123^I)FP-CIT reporting. Two contrasting datasets are used in this study, one based on historical clinical data from a single hospital and the other based on research data acquired from a number of other hospitals under a different acquisition protocol (downloaded from the Parkinson’s Progression Markers Initiative (PPMI) website, http://www.ppmi-info.org/). By selecting two contrasting cohorts, findings provide evidence of the impact of CADx beyond a single set of specific acquisition conditions and patient characteristics.

## Methods

### Automated classifier

In this study, a simple machine learning methodology was adopted for creation of classifiers, which has shown high performance in previous tests. Briefly, the algorithm consisted of a linear support vector machine (SVM) with input features derived from the first five principal components of image voxel intensities (ordered according to reducing variance) and patient age. Spatial and intensity normalisation was applied to images before training the algorithm. Spatial normalisation was achieved through multi-stage, automated, affine registration, and intensity normalisation was achieved by dividing all voxel intensities by the mean value in the occipital lobe (see [3], algorithm ML 2 for more details). An appropriate value for the ‘C’ hyperparameter in the SVM algorithm was selected through initial repeated, 10-fold cross-validation. Algorithm training was completed using Matlab software (Matlab, Natick, USA) and the libSVM library [10].

### Data

Three hundred fifty-nine historical (^123^I)FP-CIT datasets were extracted from the archives at Sheffield Teaching Hospitals NHS Foundation Trust, for patients scanned between May 2007 and May 2015, after excluding images where significant vascular disease was identified in concomitant MRI brain scans, or where the images contained significant artefacts. All patient images were acquired from dual-headed gamma cameras, manufactured by GE (3 GE Infinia and 1 GE Millenium, GE Healthcare, Chicago, USA), and all reconstructions were performed using the same GE Xeleris v2.1 software (GE Healthcare, Chicago, USA) and settings (ordered subset expectation maximisation with two iterations, 10 subsets and no scatter or attenuation correction). See Table 1 for a summary of the key patient preparation and image acquisition parameters.

**Table 1:** Summary of the acquisition and patient preparation parameters for the local and PPMI databases

Parameter	Local database	PPMI database
Administered activity	167–185 MBq	111–185 MBq
Injection-to-scan delay	3–6 h	3.5–4.5 h
Acquisition time	30 min	30–45 min
Acquisition pixel size	3.68 mm	Variable (scanner dependent)
Number of projections	120 (over 360°)	90 or 120 (over 360°)
Energy window	159 keV ± 10%	159 keV ± 10% and 122 keV ± 10%
Collimator	LEHR	Variable (scanner dependent)

The patient notes associated with the datasets, if available, were examined by two neurologists to establish a reference diagnosis. Of the examined notes, there were 55 patients for which a clinical diagnosis could be established with high confidence, based on the Queen Square Brain Bank criteria for the diagnosis of PD. Thirty-three of these were classified as having pre-synaptic dopaminergic deficit (PDD) and 22 as not having PDD. The mean time of follow-up post SPECT imaging was 31 months, with a minimum of 15 months and a maximum of 51 months. There were 34 male and 21 female patients in this subset. At the time of scanning, their mean age was 66 years (range 29–80 years). These cases were used for evaluating diagnostic performance in the reporting study only. The remaining 304 cases, without a reference clinical diagnosis, were divided into broad categories according to the original image report (113 patients without PDD and 191 with PDD; see [3] for more details) and used purely for algorithm training (classifier 1). Thus, there was a difference in the labelling methodology for different subsets of the local data, with algorithm training relying on data with a reference diagnosis based on visual assessment only, and reporting performance measured with data that had reference diagnosis based on clinical follow-up.

In addition, all the baseline (^123^I)FP-CIT examinations from the Parkinson’s Progression Markers Initiative (PPMI) database were also downloaded, for which a reference clinical diagnosis was available in all cases (209 healthy controls (HC), 448 with Parkinson’s disease (PD)). This data was used to train and evaluate a separate classifier (classifier 2), to provide additional insight into the potential impact of CADx. This classifier used the same model, hyperparameter and training procedures as classifier 1, but with the different training data can be considered a distinct algorithm. Image acquisition, reconstruction and processing parameters for the PPMI dataset are described elsewhere [11, 12]. However, the key patient preparation and acquisition parameters are summarised in Table 1. In contrast to the local data, PPMI images were reconstructed with HOSEM software (Hermes Medical, Stockholm, Sweden) utilising an OSEM algorithm with eight iterations and eight subsets and attenuation correction based on Chang’s method [13]. No scatter correction was performed.

The PPMI data were split into training and test subsets in such a way as to skew the test data towards more difficult cases. This was done due to the fact that patients were only included in the PD group if their SPECT scans displayed abnormal appearances, as assessed by the PPMI core lab team. PD patients for whom the SPECT scan was normal or equivocal were excluded from the database. This screened collection of data was therefore likely to be associated with higher visual reporting accuracy as compared to the local clinical data. To counteract this bias, more challenging cases were preferentially selected for the test set, using striatal binding ratio results as a surrogate marker of the likely difficulty in classifying the data.

The PPMI data was split in half, maintaining the same HC to PD ratio in both subgroups. The first half (328 patients) was used to train the classifier (classifier 2). For the second half of the data, semi-quantification figures were examined to find the 40 healthy controls with the lowest putamenal striatal binding ratios (SBRs) and the 60 PD cases with the highest SBRs. This collection of 100 images, skewed towards more equivocal data (according to semi-quantification results), was used in the clinical evaluation. The remaining data, which was neither used for algorithm training nor for testing with radiologists, was excluded.

### Reporting software

**Fig. 1:**
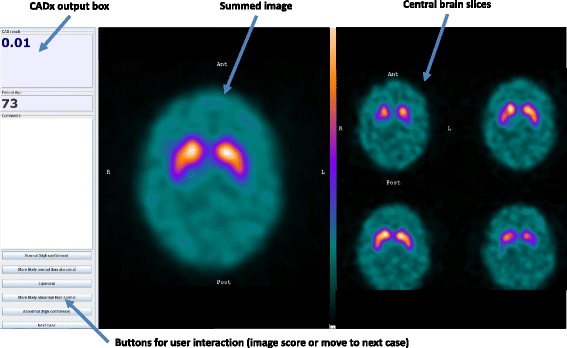
Display presented to reporters. Screenshot of the display presented to reporters during the study (in this case, the CADx output is visible)

Routine reporting in Sheffield involves visual evaluation of four reconstructed slices (7.4 mm thick) from the centre of the rigidly registered brain and of a summed image created by combining these axial slices. This data is observed using Jview (Link Medical, Bramshill, UK), a clinical platform based on Java software. An additional Java applet was written to augment the functionality of Jview, to force each set of patient images in the study to be viewed in a standard format. An additional pane was inserted on the left hand side of the screen, which contained buttons allowing the user to move to the next case or to input their diagnostic confidence score. This pane was also used to display the output from the CADx tool (at an appropriate stage of the study) and patient age (see Fig. 1).

The CADx output was in the form of a probability value. libSVM’s inbuilt function for converting SVM scores to probabilities was adopted for the chosen classification algorithms in this study, which utilises cross-validation to fit the available data to a logistic function. The probability of belonging to the abnormal class was estimated for all the patients. Given that the classifiers were binary, for cases where *P* ≥ 0.5, the corresponding probability of belonging to the normal class was 1-P (i.e. less than 0.5). In these cases, the CADx output value was displayed in red font. For patients where *P* < 0.5, the corresponding probability of belonging to the normal class was greater than 0.5 and a blue font was used in the display.

### Reporting methodology

**Fig. 2:**
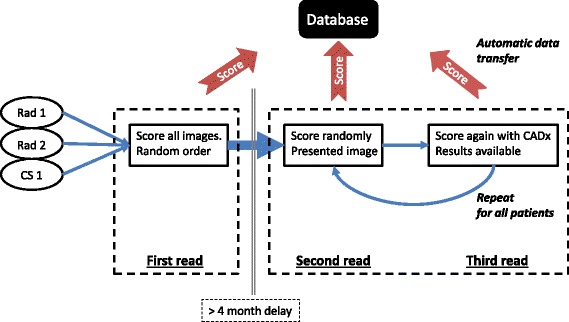
Overview of study methodology. Repeated for both the local and PPMI data

The study involved three reporters examining test images, presented in a random order, three times (an overview of the study methodology is shown in Fig. 2). On the first two occasions, reporters were asked to independently score all images in the cohort according to their confidence in either a normal or abnormal classification, through visual assessment alone. The second read commenced once all cases in the cohort had been scored once and a subsequent delay time of at least 4 months had expired. The delay between reads 1 and 2 reduced the effects from recall bias. In contrast, the second and third reads were carried out together such that immediately after a reporter had recorded a score for a particular image, they were then presented with the same image, but with the probability value from the automated classifier displayed prominently on the screen. Thus, comparison between the first and (delayed) second visual reads provided an insight into intra-reporter reliability. Comparison of the second and third reads gave an indication of the impact of CADx on reporting.

A 5-point diagnostic confidence scale was used throughout, where a score of 1 was equivalent to having high confidence that the image showed abnormal dopaminergic function and a score of 5 was equivalent to having high confidence that the image was normal. Scores of 2 and 4 were assigned to images where reporters were less confident in their overall assessment but still favoured one of the binary choices, and a score of 3 was used for any equivocal cases.

This process was repeated for both the local and PPMI datasets. Three reporters were recruited, two consultant radiologists (Rad 1 and Rad 2) and one clinical scientist (CS 1), all with greater than 5 years experience of reading (^123^I)FP-CIT images as part of routine clinical duties in a large teaching hospital. Each reporter used a clinical workstation for viewing the images, in isolation from the other reporters.

The metrics selected to evaluate reporter performance were sensitivity, specificity and diagnostic accuracy. These metrics were calculated by compressing the submitted confidence scores into three classification categories: with disease, without disease and equivocal. In addition, inter- and intra-reporter reliability were assessed using the intraclass correlation coefficient (ICC), calculated from the raw diagnostic confidence scores. ICC is a commonly applied metric for evaluating intra- and inter-rater reliability using ordinal or interval rating scales. Values of ICC can range from 0 to 1 where 1 represents perfect reliability with no measurement variability and 0 is representative of no reliability. In this study, the two-way random model was implemented for measuring inter-reporter reliability, with single measures (i.e. ICC (2, 1)), and the one-way random model with single measures (i.e. ICC (1, 1)) implemented for assessing intra-reporter reliability. These particular forms of ICC were selected based on the guides provided by Rankin [14] and Koo [15].

In addition to tests of reporter performance, the standalone accuracy, sensitivity and specificity of the CADx tool was also measured for all the test cases. This was done to confirm that the algorithm was sufficiently accurate to be used as a reporting assistant and to quantify the performance gap between the human reporters and the software.

After the study had been completed, each reporter was asked a series of set questions from a questionnaire in separate interviews. This aspect of the study was primarily designed to provide an insight into the CADx-radiologist relationship, to assess the effects of the CADx software on clinician decision-making; this is an important topic that has largely been ignored by researchers [16]. The questions included a mix of open and closed queries. Restricted response categories were included, where possible, to allow for more straightforward analysis.

## Results

**Fig. 3:**
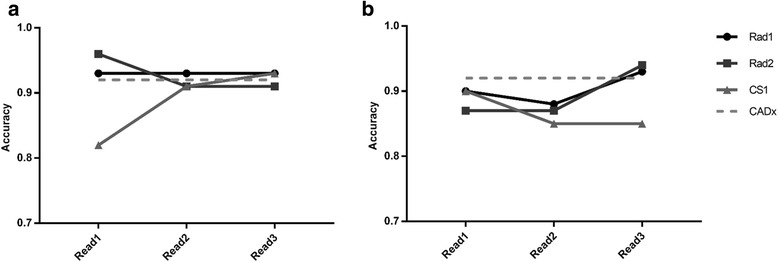
Diagnostic accuracy figures for the the image reads. Diagnostic accuracy figures for the three image reads, for local data (**a**) and PPMI data (**b**). Standalone CADx performance is also shown, for comparison

**Fig. 4:**
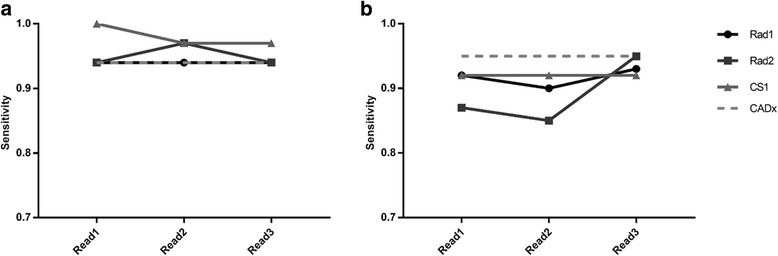
Sensitivity figures for the three image reads. Sensitivity figures for the three image reads, for local data (**a**) and PPMI data (**b**). Standalone CADx performance is also shown, for comparison

**Fig. 5:**
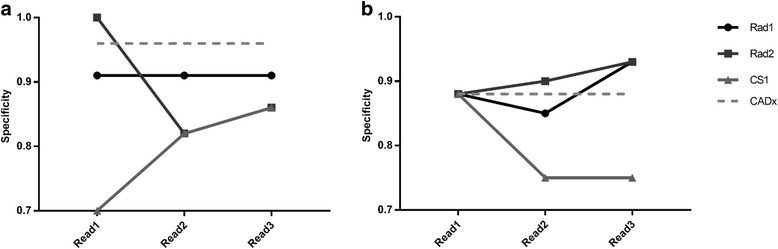
Specificity figures for the three image reads. Specificity figures for the three image reads, for local data (**a**) and PPMI data (**b**). Standalone CADx performance is also shown, for comparison

Figures 3, 4 and 5 summarise performance metrics for each reporter for each of the three reads, for local data and PPMI data respectively. Standalone performance of the CADx tool is also shown. The time delay between reads 1 and 2 ranged from 137 to 356 days across the two datasets and three reporters, well in excess of 4 months.

**Fig. 6:**
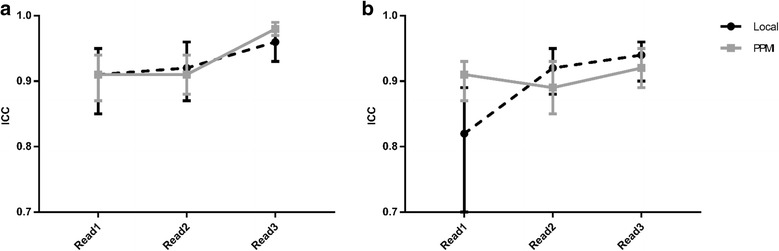
Inter-reporter reliability (ICC) results for each of the three image reads. Inter-reporter reliability (ICC) results for each of the three image reads for PPMI data and local data. Graph (**a**) is derived from radiologist data only (Rad1 and Rad2); graph (**b**) is from all reporters. Whiskers represent 95% confidence intervals

Reporters’ confidence scores changed in approximately 13% of cases for the local data and in approximately 17% of cases for the PPMI data after being exposed to the CADx software output (i.e. comparing reads 2 and 3). Intra- and inter-reporter reliability results are shown in Table 2 and Fig. 6. Separate inter-reporter reliability figures are displayed considering all three reporters together, then considering just the radiologists alone.

**Table 2:** Intra-reporter reliability (ICC) results for all reporters, with 95% confidence intervals (CI), for PPMI data and local data

	Intra-reporter reliability					
	PPMI			Local		
Reporter	ICC	95% CI (lower)	95% CI (upper)	ICC	95% CI (lower)	95% CI (upper)
Rad1	0.87	0.82	0.91	0.89	0.82	0.93
Rad2	0.95	0.92	0.96	0.93	0.88	0.96
CS1	0.91	0.87	0.94	0.88	0.80	0.93

Table 3 summarises responses received to the main questions in the questionnaire.

**Table 3:** Summary of responses to the questionnaire (restricted response categories only)

Question	Responses				
	A lot	Moderately	A little	Not at all	Unsure
In general, how well did your initial reporting decisions correlate with the CADx output?	Rad1	–	–	–	–
	Rad2				
	CS1				
	Substantial impact	Moderate impact	Small impact	No impact	Unsure
In general, how would you rate the impact of the CADx algorithm on your reporting decisions?	–	Rad1	Rad2	–	–
			CS1		
		CADx	Semi-quant	Both	Unsure
Would you prefer to have CADx for assistive DaTSCAN reporting or semi-quantification? Or Both?		–	–	Rad1	–
				Rad2	
				CS1	
	Yes (substantial benefit)	Yes (moderate benefit)	Yes (small benefit)	No	Unsure
Would it benefit you if the CADx system also provided information on how it came to its decision (e.g. reduced putamen uptake, high background uptake)	–	CS1	Rad1	–	–
			Rad2		
	Substantial benefit	Moderate benefit	Small benefit	No benefit	Unsure
To what extent would the CADx system be a useful training tool to improve DaTSCAN reporting performance for inexperienced clinicians?	Rad2	Rad1	–	–	CS1

## Discussion

This work considered the use of automated classifiers as a computer-aided diagnosis tool for (^123^I)FP-CIT imaging. Analysis of Figs. 3, 4 and 5 suggests that there was relatively high variation in reporters’ performance metrics between the first and second reads in some cases, for both sets of data. For instance, the diagnostic accuracy of CS1 changed from 0.82 to 0.91 when reporting the same set of local images. This suggests that there is a reasonable degree of intra-reporter variability when analysing images visually, even for experienced reporters. These findings were unexpected and may be related to the fact that there was a relatively long time gap between image reads, such that reporters’ impressions of what constitutes a normal or abnormal image may have drifted. Patient age was not displayed to reporters during read 1 but was available during reads 2 and 3. This may also have introduced additional variability. However, such variability may be an exaggeration of what is normally expected in the local clinical service, where a group reporting scenario is used routinely, with semi-quantitative results available. This may help to ameliorate the effects of individuals’ changing visual impression. Nonetheless, results do provide a reminder that human perception and understanding of medical images is not a constant and invites speculation that it could be improved through routine use of assistive software.

The increased consistency offered by CADx tools is demonstrated by inter-reporter reliability results. Figure 6a demonstrates that for the two radiologists at least, there was a noticeable increase in the intraclass correlation coefficient between reads 2 and 3, showing that there was reduced variability in submitted confidence scores. For the PPMI data, the 95% confidence interval bounds indicate that the increase in reliability was statistically significant. These trends are reinforced by percentage agreement figures: for the PPMI data, the radiologists had completed agreement in confidence scores in 77 and 74% of cases for reads 1 and 2, rising to 87% agreement after introduction of CADx. However, the upward trend in ICC figures is less clear when the clinical scientist was included in the analysis (see Fig. 6b).

Given the increased consistency between reporters during read 3, in terms of their confidence in a particular classification, it is likely that the introduction of a CADx system would also have benefits in terms of reduced intra-reporter variability. However, estimation of such an effect would require that the reporting exercise, with CADx assistance, be repeated.

Comparing reads 2 and 3 (i.e. directly before and after the CADx was shown to the reporter), there is evidence of some uplift in performance for the PPMI data, where accuracy, sensitivity and specificity either stayed the same or increased for all reporters. Conversely, for the local data, there was no clear change in performance as a result of the introduction of CADx. These contrasting results for the two different datasets could be partly related to the reliability of the reference diagnoses for the two different datasets. Classifier 1 was trained with (local) data where the reference classification was derived from the original image report, created through reporters’ visual analysis of the SPECT data (with patient notes and other imaging available). Thus, the CADx tool was trained to the diagnostic performance level of conventional reporting methods. Conversely, classifier 2 was trained with PPMI data where the diagnoses of the patients was better established and was not solely reliant on the (^123^I)FP-CIT scan result. In this case, standalone performance of the algorithm could have exceeded that achievable through visual interpretation, increasing the chances of CADx having a significant impact on reporters’ decisions.

For the PPMI data, it is again interesting to note the contrasting performance results between the clinical scientist (CS1) and the two radiologists (Rad1 and Rad2). Further analysis of the data suggests that CS1 only changed his confidence score in 7% of cases for the PPMI data after viewing CADx results, as compared to 21 and 22% for Rad1 and Rad2 respectively. A similar but less marked trend was seen in the local data, where CS1 changed his score in 6% of cases as compared to 9 and 23% for Rad1 and Rad2 respectively. This is consistent with the radiologists relying more heavily on the CADx decision than the clinical scientist, particularly for the unfamiliar PPMI data.

In this study, the PPMI test data was deliberately skewed towards more difficult cases in order to maximise the opportunity for CADx to influence results. This was necessary because of the strict patient group definitions set out in the PPMI protocol. In particular, scans without evidence of dopaminergic deficit (SWEDD), where patients display features associated with PD but have normal SPECT scan appearances, are classified separately to HC and PD groups. SWEDD cases were excluded from the current study, which would ordinarily lead to an increase in test accuracy beyond that which might be expected in clinic. For illustrative purposes, applying classifier 2 to the 76 SWEDD cases in the PPMI database gives an abnormal classification in only 7 of 76 patients (the remaining 69 cases are classified as belonging to the non-diseased group).

The effects of skewing the PPMI test database can be demonstrated through analysis of standalone CADx and semi-quantitative performance figures. In previous work [3], it was shown that a classifier based on five principal components and a linear SVM achieved a mean diagnostic accuracy of 0.97 for randomly sampled data, the joint highest performance of all the machine learning methods considered. In the current study, accuracy was lower, i.e. 0.92 for the skewed PPMI data. Similarly, a semi-quantitative method based on finding the optimum point on an ROC curve of putamen uptake values (SQ 17 in [3]), gave a mean accuracy value of 0.95 for randomly selected PPMI test data [3]. This was found to be the best performance achieved of all the tested semi-quantitative approaches. However, in the current study, the performance for the same method dropped to 0.74 for skewed PPMI data. Thus, by manipulating the PPMI data, results demonstrate that it was possible to reduce algorithm accuracy, by implication making the data more difficult to interpret by reporters.

It is difficult to directly compare findings of the current study to those of studies evaluating the effects of semi-quantification on radiologists’ performance, mainly due to differences in data used and methodology. However, the broad findings of this work—that CADx can improve accuracy if adopted by reporters with limited experience of the data and that consistency in terms of diagnostic confidence scores may also improve as a result—are similar to much of those of the previous work related to semi-quantification [4–9].

These broad similarities are perhaps surprising given that machine learning algorithms have previously been shown to differentiate themselves from a wide range of semi-quantitative methods in terms of standalone performance, albeit by a small margin in most cases [3]. Thus, although the CADx system used here offers advantages over conventional semi-quantification approaches, questions remain as to whether this translates into improved clinical performance above and beyond that offered by semi-quantification.

Evaluation of the radiologist-CAD relationship is rarely carried out. In this study, the questionnaires provided to participating radiologists give a useful insight into CADx’s influence on decision-making and how it could be improved. The responses suggested that the CADx tool generally agreed well with the reporters’ classification decisions, with only a very limited number of disagreements. This reflects the quantitative analysis above. The classifiers mostly had a small or moderate impact on decision-making processes, which was as expected for an application where normal and abnormal appearances are often relatively easy to identify. The most common comment was that the CADx tool gave reporters added confidence in their decision, in a similar way to what might be expected from the presence of a human second reader.

Interestingly, all three reporters felt that having access to both CADx and semi-quantification was preferable to having access to one or the other. This implies that the functionality of each was felt to be positive and complementary. It might be speculated that a greater impact on reporting performance can be measured by performing a clinical study using a combined software algorithm that outputs both striatal binding ratios and overall probabilities.

The questionnaire results provide additional evidence that the approaches and opinions of the two radiologists were close to each other but differed from that of the clinical scientist. In general, the clinical scientist was less positive about the CADx tool and more cautious about relying upon it.

The testing scenario was associated with some limitations. As mentioned previously, patients’ clinical history was not available to reporters as it would have been in clinic. If such information were available, the impact of CADx may have been different. However, machine learning algorithms can also make use of clinical history data, and the addition of these inputs may help to rebalance relative performance. Secondly, patient age was only provided to reporters on reads 2 and 3. This may have caused additional intra-reporter variability. Even so, the data implies that the impact of CADx for the radiologists was at least as big as any differences in reporting performance attributed to inclusion/exclusion of patient age.

The reference diagnoses of all the images studied was binary (i.e. either with or without disease). However, the 5-point confidence scale used by reporters associated a score of 3 with an equivocal classification, giving users a choice of three different classifications. This mismatch dictated that accuracy, sensitivity and specificity were all negatively affected whenever a reporter submitted an equivocal confidence score. Although a score of 3 was selected in less than 3% of cases, this suggests that metrics of diagnostic performance may be more pessimistic than might have been the case if only two classifications were available for users to select. The diagnostic confidence scores reported are likely to be closely correlated with disease severity. However, it should be emphasised that these are distinct concepts. If a disease severity scale had been provided to reporters, the intra- and inter-operator variability results may have been slightly different.

In respect of wider application, this study examined two classifiers (classifiers 1 and 2) trained separately with data from distinct sources. There may be a negative impact on classifier performance should the algorithms be applied to data acquired from different equipment, in different institutions. Indeed, the classifier calibrations applied to convert classifier outputs into probabilities may be misleading under these circumstances. If machine learning tools are to be used more widely, these issues require further investigation.

## Conclusions

This study represents a comparative diagnostic exercise involving identification of patients with pre-synaptic dopaminergic deficit for two sets of data (local, PPMI) using established and CADx reporting methods. The performance of all the experienced reporters demonstrated a degree of variability when analysing images through visual analysis alone. However, inclusion of CADx improved accuracy, sensitivity and specificity for two experienced radiologists, when viewing (unfamiliar) PPMI data.

In addition, the introduction of CADx increased consistency between the two radiologists, in terms of their diagnostic confidence scores, for both the PPMI and local data. Clinical scientist reporting performance was less affected by the CADx tool with little change in reporting performance between reads 2 and 3, for both sets of patient images. The more cautious approach of the clinical scientist was also evident in responses to the questionnaire, which sought to assess usability of the tool. These qualitative results also revealed that all reporters would prefer to have access to both semi-quantification and CADx in clinic, rather than one or other in isolation. The outcomes for this study indicate the value of CADx as a diagnostic aid in the clinic and encourage future development for more refined incorporation into clinical practice.

## Abbreviations

CADx: Computer-aided diagnosis
CI: Confidence interval
HC: Healthy control
ICC: Intraclass correlation coefficient
MSA: Multiple system atrophy
PD: Parkinson’s disease
PDD: Pre-synaptic dopaminergic deficit
PPMI: Parkinson’s Progression Markers Initiative
PSP: Progressive supranuclear palsy
ROC: Receiver operator characteristic
SBR: Striatal binding ratio
SPECT: Single-photon emission computed tomography
SVM: Support vector machine

## References

[1] Palumbo B, Fravolini ML, Nuvoli S, Spanu A, Paulus KS, Schillaci O (2010). Comparison of two neural network classifiers in the differential diagnosis of essential tremor and Parkinson’s disease by (123)I-FP-CIT brain SPECT. Eur J Nucl Med Mol Imaging.

[2] Huertas-Fernández I, García-Gómez FJ, García-Solís D, Benítez-Rivero S, Marín-Oyaga VA, Jesús S (2015). Machine learning models for the differential diagnosis of vascular parkinsonism and Parkinson’s disease using [(123)I]FP-CIT SPECT. Eur J Nucl Med Mol Imaging.

[3] Taylor JC, Fenner JW (2017). Comparison of machine learning and semi-quantification algorithms for (I123)FP-CIT classification: the beginning of the end for semi-quantification?. EJNMMI Physics.

[4] Skanjeti A, Angusti T, Iudicello M, Dazzara F, Delgado Yabar GM, Trevisiol E (2014). Assessing the accuracy and reproducibility of computer-assisted analysis of (123) I-FP-CIT SPECT using BasGan (V2). J Neuroimaging.

[5] Soderlund TA, Dickson J, Prvulovich E, Ben-Haim S, Kemp P, Booij J (2013). Value of semiquantitative analysis for clinical reporting of I-123-2-beta-carbomethoxy-3 beta-(4-iodophenyl)-N-(3-fluoropropyl)nortropane SPECT studies. J Nucl Med.

[6] Albert NL, Unterrainer M, Diemling M, Xiong G, Bartenstein P, Koch W (2016). Implementation of the European multicentre database of healthy controls for [(123)I]FP-CIT SPECT increases diagnostic accuracy in patients with clinically uncertain parkinsonian syndromes. Eur J Nucl Med Mol Imaging.

[7] Booij J, Dubroff J, Pryma D, Yu JQ, Agarwal R, Lakhani P, et al. Diagnostic performance of the visual reading of (123)I-ioflupane SPECT images when assessed with or without quantification in patients with movement disorders or dementia. J Nuclear Med. 2017; 10.2967/jnumed.116.189266. 10.2967/jnumed.116.189266 28473597

[8] Ueda J, Yoshimura H, Shimizu K, Hino M, Kohara N (2017). Combined visual and semi-quantitative assessment of (123)I-FP-CIT SPECT for the diagnosis of dopaminergic neurodegenerative diseases. Neurol Sci.

[9] Pencharz DR, Hanlon P, Chakravartty R, Navalkissoor S, Quigley A-M, Wagner T (2014). Automated quantification with BRASS reduces equivocal reporting of DaTSCAN (123I-FP-CIT) SPECT studies. Nuclear medicine review Central & Eastern Europe.

[10] Chang C-C, Lin C-J (2011). LIBSVM: a library for support vector machines. ACM Trans Intell Syst Technol.

[11] Wisniewski G, Seibyl J, Marek K (2013). DatScan SPECT image processing methods for calculation of striatal binding ratio. Parkinson’s Progression Markers Initiative.

[12] The Parkinson Progression Marker I (2010). Imaging technical operations manual. The Parkinson Progression Marker Initiative.

[13] Chang LT (1978). A method for attenuation correction in radionuclide computed tomography. IEEE Trans Nucl Sci.

[14] Rankin G, Stokes M (1998). Reliability of assessment tools in rehabilitation: an illustration of appropriate statistical analyses. Clin Rehabil.

[15] Koo TK, Li MY (2016). A guideline of selecting and reporting intraclass correlation coefficients for reliability research. J Chiropr Med.

[16] Eadie LH, Taylor P, Gibson AP (2012). Recommendations for research design and reporting in computer-assisted diagnosis to facilitate meta-analysis. J Biomed Inform.

